# Apparent attenuation by opto-acoustic defocus in phonon microscopy

**DOI:** 10.1016/j.pacs.2020.100180

**Published:** 2020-05-17

**Authors:** Fernando Pérez-Cota, Salvatore La Cavera III, Shakila Naznin, Rafael Fuentes-Domínguez, Richard J. Smith, Matt Clark

**Affiliations:** Optics and Photonics Group, Faculty of Engineering, University of Nottingham, University Park, Nottingham, NG7 2RD, UK

**Keywords:** Sound attenuation, Picosecond laser ultrasound, Ultrafast ultrasonics, Time-resolved Brillouin Scattering

## Abstract

Understanding the mechanical properties of biological cells is a challenging problem for the life sciences partly because there are limited methods for mapping elasticity with high resolution. Phonon microscopy is a form of Brillouin light scattering which uses coherent phonons for imaging with elasticity-related contrast, phonon resolution and without labels. It can measure material properties such as sound velocity, acoustic impedance and attenuation. To use it as a contrast mechanism in microscopy, high numerical aperture (NA) lenses are key to high resolution. However, increasing NA induces *apparent attenuation*, a premature decay of the detected signal. To reduce signal decay and quantify the sound attenuation coefficient in cells, it is necessary to understand the mechanisms that affect signal decay. Here we define *opto-acoustic defocus* as a signal decay mechanism and propose methods to achieve quantitative sound attenuation measurements, and to optimise in-depth imaging at high resolution which is crucial for cell imaging.

## 1 Introduction

Elasticity is emerging as an important parameter for the characterisation of biological materials. Numerous works have demonstrated the ability to use elasticity measurements as a way to characterize biophysical parameters [1], [2], [3], [4], [5]. Among the available techniques for elasticity characterisation, those based on Brillouin light scattering (BLS) are particularly promising because they offer a non-invasive measure of the product of the refractive index and the speed of sound with optical resolution [6], [7], [8], [9], [10], [11].

Phonon microscopy is a novel technology that uses coherent phonon fields to image biological cells with contrast provided by BLS [9], [12]. Through the use of ultra-fast pump-probe methods [13], the technique provides access to the phenomena of BLS in picosecond time scales (time-resolved Brillouin scattering (TRBS)). An interesting consequence of the high temporal resolution phonon microscopy provides, is that material properties such as sound velocity, acoustic impedance and sound attenuation can be measured directly in the time domain with sub-optical resolution provided by the phonon wavelength [14].

Sound attenuation is the loss of energy of a propagating sound wave and is a relevant characteristic of materials [15], [16], [17], [18], [19]. For a non-diffracting plane wave, this loss is related to properties of the medium such as viscosity, inhomogeneity and the frequency of the wave. The relationship of these properties with the attenuation of sound are not easy to model, especially for the case of heterogeneous biological materials. Nevertheless, in cancer research sound attenuation has been used as a biomarker to characterise cells and tissue; for instance, it has been used to image uterine [20], [21] and breast tumours [22], as well as to identify cancer in fluid [5].

Imaging cells at high resolution with contrast given by sound attenuation offers an opportunity for applications in cell biology at the single cell level. However, imaging with high lateral resolution requires high numerical aperture (NA) lenses to reduce the size of the illumination spot. However, increasing NA has the potential to introduce a parasitic source of signal decay which is unrelated to the elastic properties of the specimen. Such an effect would have two important implications for phonon imaging: reduction of measuring depth and errors on the quantification of the sound attenuation coefficient (*α*_0_).

The effect of NA has been reported extensively for spontaneous Brillouin spectroscopy (SBS) [23], [24], however for TRBS, the conditions are different. Whereas in SBS, the symmetry of the Brillouin frequency spectrum is in part determined by the range of spatial frequencies produced by the NA of the objective lens, in TRBS this is less relevant as the strong coherence of the longitudinal phonons enhances scattering of the lower spatial frequencies (parallel to the optical axis). Nevertheless, effects of NA in TRBS have been reported [16] yet not thoroughly investigated, and have been avoided by either using a pulse-echo approach [25] or using low NA lenses [26]. More recently, their cause has been attributed to acoustic diffraction [27].

In this article we report the loss of signal with increasing NA and the measurement of the attenuation coefficient as performed by TRBS. Based on model and experimental results, it is concluded that, contrary to previous reports, a main cause leading to an apparent increase in the attenuation coefficient is *opto-acoustic defocus*, which occurs when the coherent phonon field propagates beyond the Rayleigh range of the optical system. The implications of this conclusion are discussed within the context of cell imaging.

### 1.1 Sound attenuation and signal decay in phonon microscopy

**Fig. 1:**
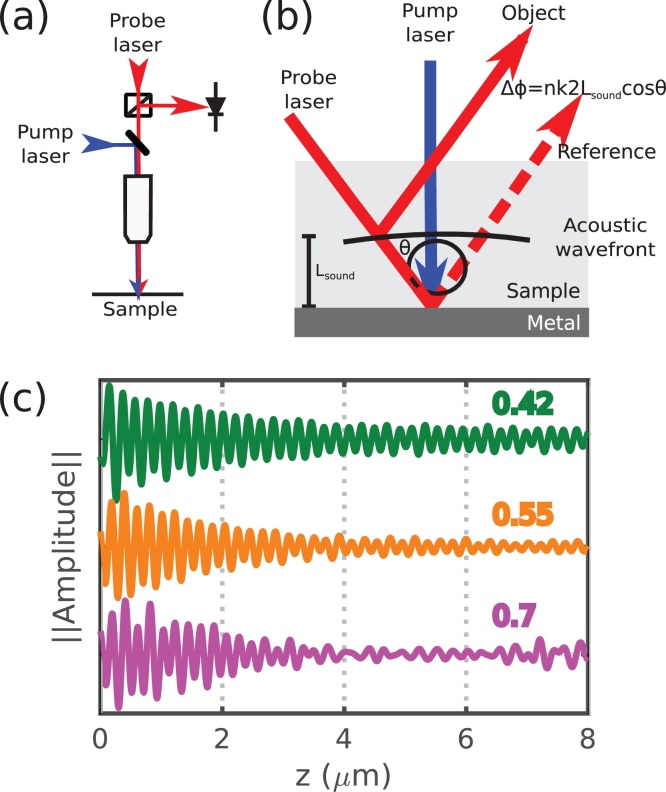
Typical decay observed in time resolved Brillouin scattering in glass using different NA lenses. (a) Typical TRBS experimental configuration. (b) Interaction of light and sound in TRBS where the phase difference between the object and reference beams is dictated by the Bragg condition. (c) Typical detected signals in glass. An increase in NA gives an apparent increase in attenuation.

TRBS is measured experimentally with a pump-probe system which uses femtosecond lasers pulses to resolve picosecond phenomena [13], [28], [29], [30]. Sound is produced by focusing a pulsed pump laser into a metallic transducer layer which launches a strain pulse due to thermal expansion (see Fig. 1). A time-delayed pulsed probe laser is focused at the same point as the pump (see Fig. 1a) and light is scattered from the wavefront of the strain pulse as it propagates axially away from the source (see Fig. 1b). The acoustic signal arises from the interference of the sound-scattered (object) light with the directly propagating (reference) light at a detector which produces an oscillatory change in the detected intensity (see Fig. 1c). The frequency of the time-resolved signal (the Brillouin frequency, *f*_*B*_) is a measurement that is proportional to the sound velocity:

(1) $$f_{B}=\frac{2n\nu }{\lambda_{\mathrm{probe}}}\cos\theta$$

where *n* and *ν* are the refractive index and sound velocity of the medium respectively, *λ*_*probe*_ the optical probe wavelength in vacuum and *θ* angle of incidence. The amplitude of the TRBS signal is also directly related to the intensity of the phonon and photon fields. By tracking the frequency and decay of this signal, it is possible to measure the sound velocity and attenuation coefficient (*α*_0_) for semi-transparent and transparent materials, such as biological cells. However, in these experiments, it has been observed that signal attenuation increases with NA (see Fig. 1c) in what we call *apparent attenuation*. Previously, this effect has been attributed to acoustic diffraction [27] and little attention has been given to the contributions of the optical effects.

We propose that apparent attenuation also arises in TRBS by *opto-acoustic defocus* which occurs when the phonon wavefront (originated at the beam waist) propagates beyond the Rayleigh range of the optical system. Here, the optical beam that carries the signal, drops in intensity at the detector plane (due to the divergence of the optical beams) which causes loss of interference contrast and could be mistaken as sound attenuation (see Fig. 1c). As NA increases, this effect becomes more aggressive producing signal decay comparable, or even greater than, that produced by the combination of sound attenuation and acoustic diffraction.

## 2 Modelling

Acoustic diffraction (divergence of the acoustic field) has been previously attributed as a leading cause of apparent attenuation [27]. However we believe this is an incomplete conclusion since the near field distance of the optical wavelength actually matched that of the acoustic beam, and therefore the decay of the scattered optical intensity cannot be neglected. To individually test the contributions of the optics and acoustics to apparent attenuation, their near field distances should be varied independently. In order to achieve this, separate objective lenses with unique NA are used to generate the sound and optical fields.

We have developed a simple model that calculates the signal decay (interference contrast) as a function of the NA of the pump and probe lenses. Once the NA of each lens has been determined in the model set up, point spread functions (PSF) for the pump and probe wavelengths were calculated using an Airy disk representation (see supplement). The sound field generated at the transducer layer is proportional to the PSF of the pump beam focused at the transducer and its initial wavefront is flat. However, its wavelength is governed by the optical probing wavelength as defined by the Bragg condition for beams propagating along the same direction: λ_sound_ = λ_probe_ / 2n where *n* is the refractive index in the medium for *λ*_*probe*_ [9].

An Airy disk approximation is valid for this study since (1) the initial pump and probe fields originate from a lens (circular aperture) and propagate the respective focal distance (far field regime), (2) there are no features at the beam waist (e.g. an aperture or grating), (3) the optical polarization is constant given the lack of extreme incidence angles (e.g. at very high NA), (4) and the acoustic evanescent spatial frequencies would be twice that of the optics (due to the Bragg condition).

**Fig. 2:**
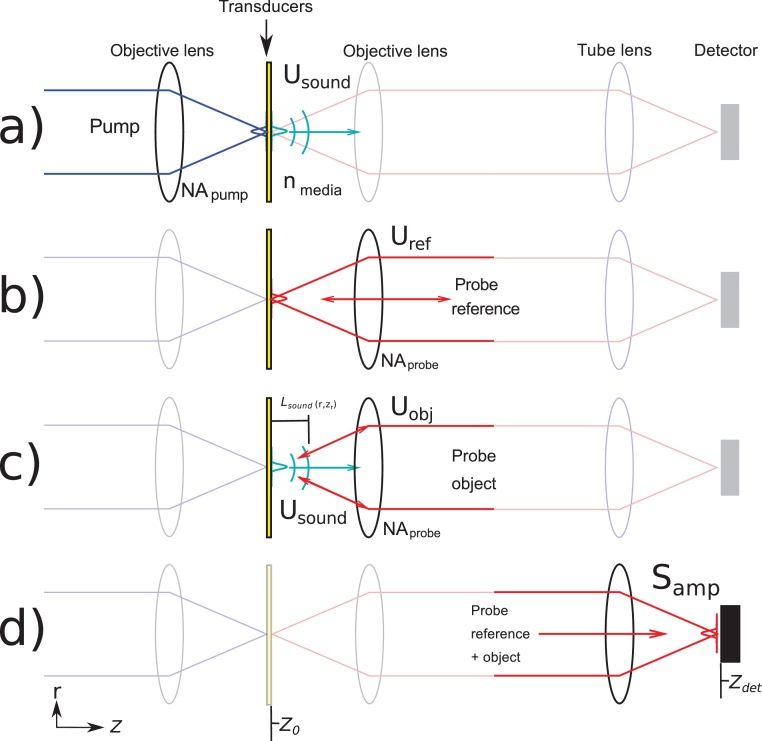
Schematic of the modelling used in this paper. The model is broken into four stages (top-bottom): (a) The pump beam PSF is computed and this defines the spatial distribution of the source of the picosecond ultrasound at the thin-film transducer layer (*z*_0_). The pump PSF was assumed to be an Airy disk and is proportional to the initial sound field which is propagated into the medium using Fourier-Bessel angular spectrum propagation (FBASP). (b) The probe (reference) beam is computed (similar to the pump) and then propagated back to the objective lens using FBASP. (c) The probe (object) beam is “scattered” off the sound wavefront and then propagated to the objective lens using FBASP. (d) The probe reference and object beams are propagated through the optical system using FBASP to the photodetector where they are “interfered” and the response on the detector is computed by integrating the light over the detector area.

From the pump and probe PSF, the initial acoustic and optical fields were defined to be *u*_*sound*_(*r*, *z*_0_) and *u*_*probe*_(*r*, *z*_0_) respectively – where *r* is the radius and *z*_0_ is the position of the beam waist and transducer–medium interface (see Fig. 2a–b). Then the optical and acoustic fields are propagated between the various planes along the optical axis to estimate field amplitude and phase using Fourier-Bessel angular spectrum propagation (FBASP) [31].

The probe light necessary to calculate signal decay at the position of the detector (*z*_*det*_) consists of two interfering fields, the probe reference beam, *u*_ref_ = *u*_*probe*_(*r*, *z*_*det*_), which is a direct reflection of the incoming probe beam from the transducer layer (see Fig. 2b) and the probe object beam, *u*_obj_(*r*, *z*_*det*_), which scatters and originates from each *r*, *z* position of the sound field (see Fig. 2c).

The amplitude of the scattered object beam (*U*_obj_(*r*, *z*)) can be modelled as being proportional to the product of the amplitudes of the probe field (*U*_*probe*_(*r*, *z*)) and the sound field (*U*_*sound*_(*r*, *z*)) as derived by Matsuda *et al* [32]. Determining the phase of the object beam (*ϕ*_*obj*_(*r*, *z*)) can be simplified by using the Bragg condition. Consequently the object photons which partake in TRBS will have phases that are a function of twice the acoustic path length (*L*_*sound*_, see Fig. 1b):

(2) $$\phi_{\mathrm{obj}}(r,z)=2nk_{\mathrm{probe}}L_{\mathrm{sound}}(r,z)=2{\mathrm{nk}}_{\mathrm{probe}}\frac{\phi_{\mathrm{sound}}(r,z)}{k_{\mathrm{sound}}}$$

where *k*_*probe*_ and *k*_*sound*_ are the optical and acoustic wavenumbers respectively and *ϕ*_*sound*_(*r*, *z*) is the phase of the acoustic field. Therefore, the object field at every position in space can be written as:

(3) $$u_{\mathrm{obj}}\left(r,z\right)=U_{\mathrm{obj}}(r,z)e^{i\phi_{\mathrm{obj}}}=U_{\mathrm{probe}}\left(r,z\right)|U_{\mathrm{sound}}\left(r,z\right)|e^{i\phi_{\mathrm{obj}}}$$

The object beam is then propagated from each *r*, *z* position through the optical system to the detector (*z*_*det*_). The objective and tube lenses were modelled by simply adding a spherical phase function to the incident wavefront. At the detector, the object and reference fields are then interfered and integrated as a function of the radius *r*. Finally, the signal amplitude is calculated as the modulation depth of the interference between the reference and object beams:

(4) $$S_{\mathrm{amp}}=\frac{2\sqrt{I_{\mathrm{ref}}I_{\mathrm{obj}}}}{\left(I_{\mathrm{ref}}+I_{\mathrm{obj}}\right)}=\frac{2U_{\mathrm{ref}}U_{\mathrm{obj}}}{\left(I_{\mathrm{ref}}+I_{\mathrm{obj}}\right)}$$

where *I*_*ref*_ and *I*_*obj*_ are the intensities of the object and reference beams respectively at the detector and *U*_*ref*_ and *U*_*obj*_ their respective amplitudes.

The NA of the pump and probe lenses are set during the calculation of the Airy disks and for the probe reference beam, this NA is preserved. However, the acoustic field can be smaller than the probe field (due to *λ*_*pump*_ < *λ*_*probe*_) and can introduce additional spatial frequencies into the object beam. Consequently, it is necessary to enforce the probe NA by introducing an aperture function at the objective lens.

We have considered a single acoustic wavelength model since the probe beam only interacts with the acoustic wavelength that satisfies the Bragg condition (see equation 1) despite the generation of a broadband pulse. Additionally the broadening of *λ*_*sound*_, given by the incident angle in equation 1, is small (∼10% in our case for NA=0.7) and does not change *u*_*sound*_ significantly. More rigorous models have been implemented [28], however in the context of probing the causes of apparent attenuation, a single wavelength model is sufficient to approximate signal decay.

In the context of using this model to test the sources of signal decay, we have not considered the effect of the limited coherence length; femtosecond laser pulses typically used in TRBS have coherence distance longer than the propagation distance of GHz sound. Additionally, the coherence distance is not a function of NA.

The model was executed with *NA*_*pump*_ and *NA*_*probe*_ as the input variables. In order to investigate how the choice of probe NA affects signal decay (opto-acoustic defocus), NA_*pump*_ was fixed to be low (0.1) while NA_*probe*_ increases. Conversely, to investigate how the choice of pump NA affects signal decay (acoustic diffraction), NA_*probe*_ was fixed (0.1) while NA_*pump*_ increases. The output of each simulation (*S*_*amp*_) was normalised to unity at *z*=0 for comparison with the experimental results.

**Fig. 3:**
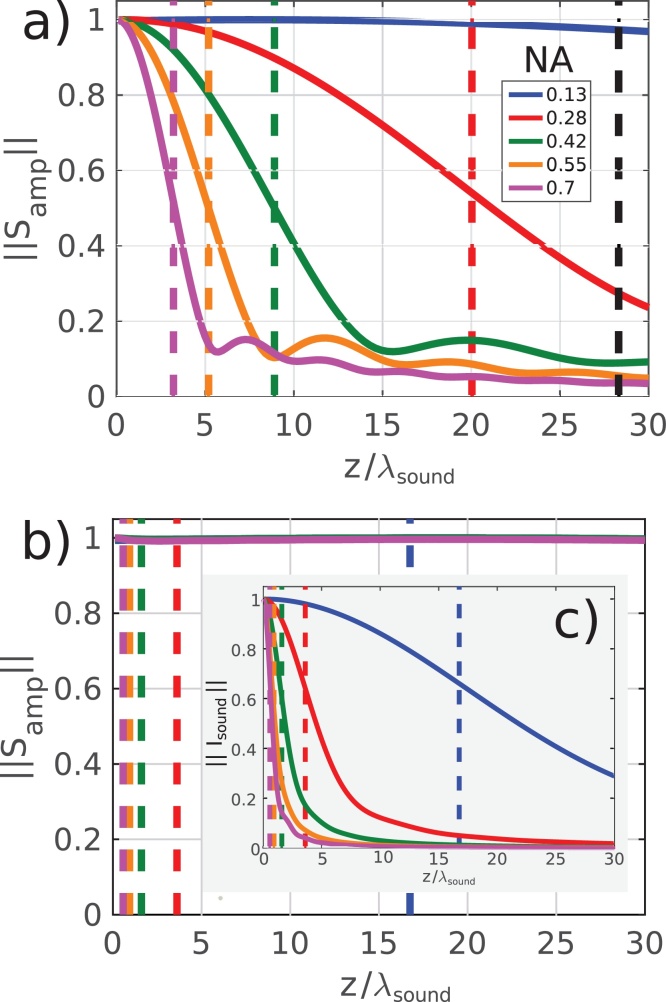
Simulated modulation depth of TRBS signals *S*_*amp*_, i.e. the apparent attenuation by (a) opto-acoustic defocus and (b) acoustic diffraction. (c) shows the drop in intensity *I*_*sound*_ along the optical axis. In (a) the vertical coloured dashed lines represent the Rayleigh range for the probe beam and the black line the Fraunhofer distance for the sound beam. In (b-c) the coloured lines represent the Fraunhofer distance for the sound beams and the Rayleigh range for the probe beam is off the scale.

The resultant S_*amp*_ traces calculated for the different combinations of NA are shown in figure 3 Figure 3. The relative change in *S*_*amp*_ due to opto-acoustic defocus is shown in figure 3a where a clear decrease is seen with *z* especially for NA_*probe*_> 0.13. The Rayleigh range for each probe NA (indicated as a coloured dashed line) occurs before the Fraunhofer distance of the sound field (black dashed line) and appears to correspond with the position at which the decay of rate of *S*_*amp*_ is maximum.

Conversely, figure 3b reveals the signal decay (*S*_*amp*_) due primarily to acoustic diffraction. Even though the sound leaves the acoustic near field (Fraunhofer distances shown in the coloured dashed lines) much earlier than the optical near field (Rayleigh range beyond the plot limit), this does not appear to affect the overall signal decay. In order to verify that the sound fields are indeed diffracting, yet the effect on the signal is not dominant, the acoustic intensity (*I*_*sound*_) as a function of *z* is presented in figure 3c. This set of simulations also represents the typical NA and propagation distances seen in previously reported TRBS contributions.

## 3 Results

**Fig. 4:**
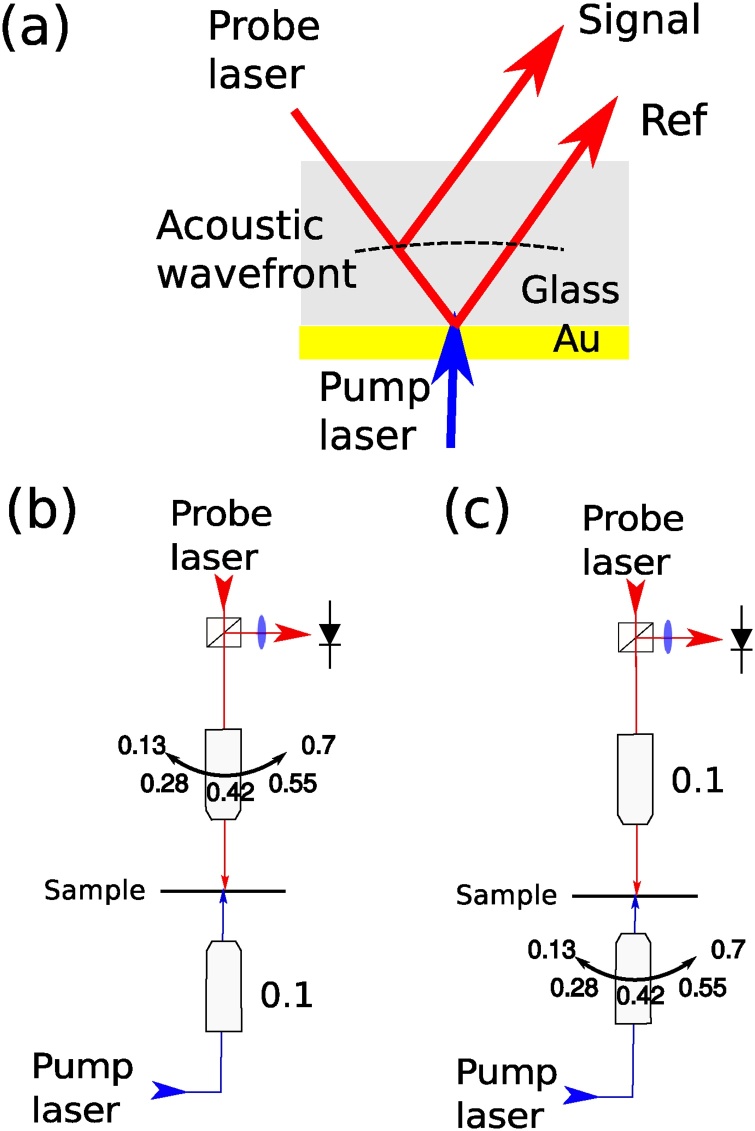
Experimental configurations for the study of apparent attenuation. (a) Beam path at sample. The pump beam is delivered from below and excites phonons in the thin film transducer. The probe beam is split at the acoustic wavefront, and its parts (signal and reference) interfere at the detector. (b) shows the configuration for the study of apparent attenuation due to opto-acoustic defocus. (c) shows the configuration for the study of apparent attenuation due to acoustic diffraction.

In order to replicate the method employed by the model, a similar strategy of varying the pump-delivery and probe-delivery/collection NA was used to experimentally probe the sources of apparent attenuation (see Fig. 4). In all cases the sample under study is a glass coverslip which is coated with 20nm of gold to act as an opto-acoustic transducer and is placed at the beam waist (Fig. 4a). The traces are obtained using a pump-probe system in an ASOPS configuration [29], with a pump wavelength of 415nm and a probe wavelength of 780nm, which corresponds to an acoustic wavelength (in glass) of ∼250nm. TRBS detection takes place by collecting the reflected signal as usually observed in previous works [13], [33], [34], however the pump beam is delivered from the opposite side with another objective. This allows changing NA_*probe*_ while maintaining NA_*pump*_ and vice versa (see Fig. 4). The position of the sample at the beam waist was corroborated by simultaneous optical brightfield illumination.

**Fig. 5:**
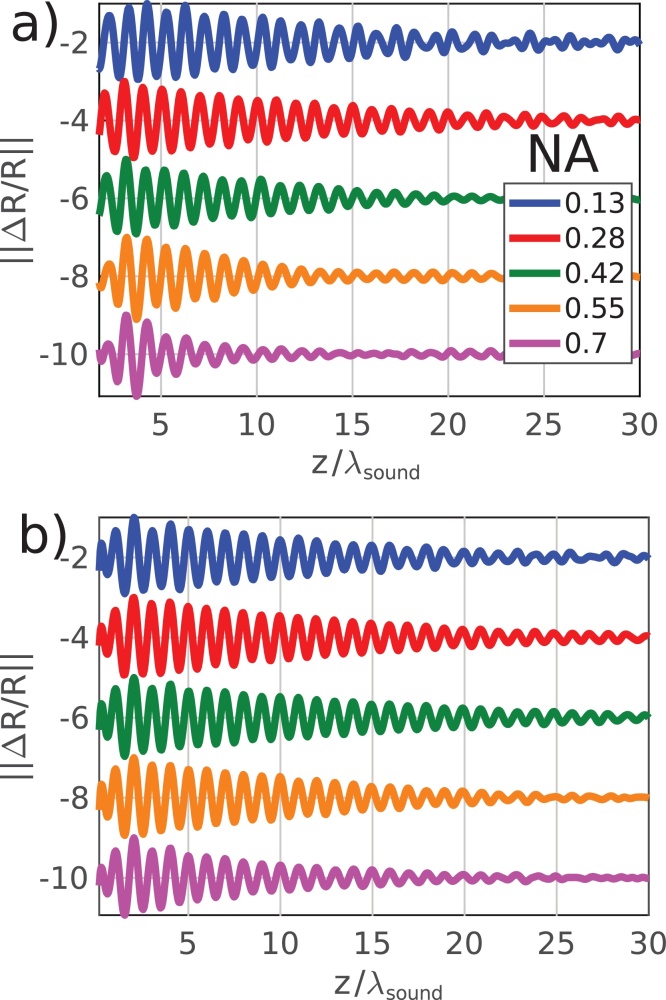
Experimental results. (a) Time traces of TRBS signals ΔR/R obtained from glass at fixed NA_*pump*_ and increasing NA_*probe*_ to observe apparent attenuation due to opto-acoustic defocus. (b) Time traces of TRBS signals obtained from a glass sample at fixed NA_*probe*_ and increasing the NA_*pump*_ to observe apparent attenuation due to acoustic diffraction. These results confirm that opto-acoustic defocus is the strongest contribution to apparent attenuation.

Experimental measurements of the TRBS signals, modulated by apparent attenuation, are presented in figure 5 Figure 5. Figure 5a corresponds with the configuration shown in figure 4b, and figure 5b with that of figure 4c. The implications of these experimental results will be discussed in the following section, but initially the contribution of opto-acoustic defocus appears to outweigh that of solely acoustic diffraction.

## 4 Discussion

Glass was used instead of water for model validation because: (a) reports of the attenuation coefficient for glass are more widely available in the literature and (b) the acoustic wavelength is similar to that of water (∼8% variation, see supplement). The attenuation of sound for a wave *S* at a position *z* is described as an exponential decay *S*(*z*) = *S*_0_(*z*)*e*^−*α*_0_*z*^ where *S*_0_ is the initial amplitude and *α*_0_ the attenuation coefficient. To validate the model and determine the attenuation coefficient of the glass sample, simulated (S_*amp*_) and experimental results (envelopes of ΔR/R) were all fitted to exponential curves to obtain their *α*_0_. The simulated modulation depths are not exponential functions, however an exponential approximation allows comparing the relative change in decay for S_*amp*_ at different NA's (see supplement). This resulted in four different attenuation coefficients: apparent attenuation by opto-acoustic defocus (*α*_*def*_) and diffraction (*α*_*dif*_) estimated by simulations, and experimental defocus ($\alpha_{\mathrm{def}}^{\prime }$) and diffraction ($\alpha_{\mathrm{dif}}^{\prime }$). The simulated attenuation coefficients do not contain the contribution of the material (*α*_0_); whereas the experimental ones are a combination of material and apparent attenuation:

$$\alpha_{\mathrm{def}}^{\prime }=\alpha_{0}+\alpha_{\mathrm{def}}\alpha_{\mathrm{dif}}^{\prime }=\alpha_{0}+\alpha_{\mathrm{dif}}$$

The attenuation coefficient *α*_0_ is then given by the offset between the simulated and experimental results:

(5) $$\alpha_{0}=\alpha_{\mathrm{dif}}^{\prime }-\alpha_{\mathrm{dif}}=\alpha_{\mathrm{def}}^{\prime }-\alpha_{\mathrm{def}}$$

**Fig. 6:**
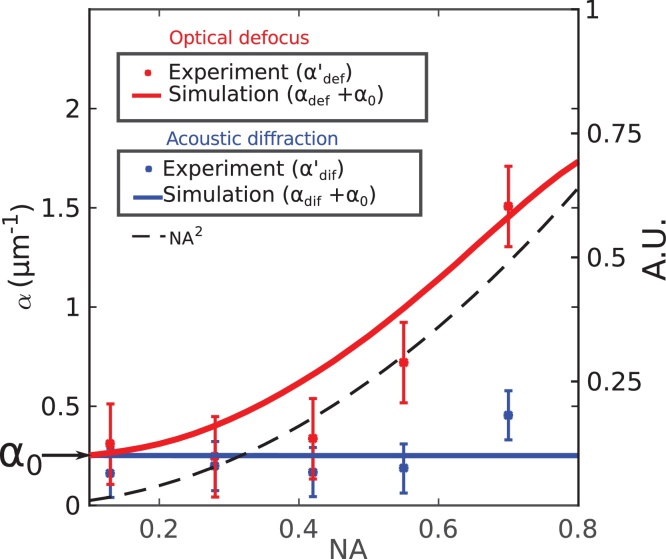
Comparison of model and experimental estimation of apparent attenuation. Results for the case of acoustic diffraction and opto-acoustic defocus are shown in blue and red respectively. The experimental and simulated results are shown in stars and solid lines respectively. The attenuation coefficient for glass calculated from equation 5 was obtained for all experimental NAs and its mean value was found to be *α*_0_= 2.5x10^5^m^−1^. The attenuation coefficient for glass was then added to the simulated results for comparison. The dotted line (NA^2^) represents an approximation to the change in depth of focus and follows the same trend as the *α*_*def*_.

Figure 6 shows the comparison of the apparent attenuation due to opto-acoustic defocus (red) and diffraction (blue) obtained from simulation (solid lines) and experiment (stars). Equation 5 was calculated for each set of experimental NAs, the average of these was taken to be the attenuation coefficient for glass, *α*_0_ = 2.5x10^5^m^−1^, which is comparable to previous reports (10^5^m^−1^ [35], [36]). In order to compare simulation with experiment, the calculated *α*_0_ was added to the simulated apparent attenuations (which did not include material attenuation contributions). Both simulation and experiment, for the case of opto-acoustic defocus, follow the shape of the inverse depth of focus (NA^2^, dotted line) corroborating that opto-acoustic defocus, an optical effect, is a significant contributor to apparent attenuation.

**Fig. 7:**
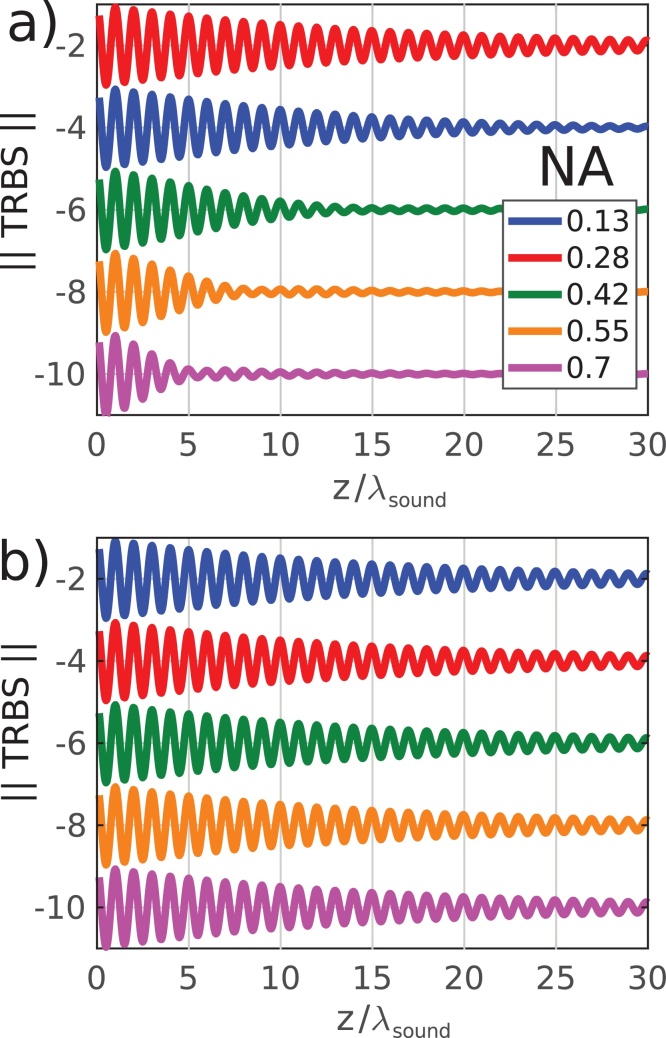
Simulated TRBS signals from glass with *λ*_*probe*_=780nm. (a) Shows the simulated effects of opto-acoustic defocus with NA. (b) Shows the simulated effects of acoustic diffraction with NA. There is a good match in the signal decay between simulation and experiment (compare with figure 5) which indicates defocus is the main cause of apparent attenuation.

Based on the measurement of *α*_0_ (glass), it was possible to produce a simulated TRBS signal using the known refractive index of glass (n=1.45@780nm [37]), for normal incidence and the measured Brillouin frequency of the form:

$$\mathrm{TRBS}\left(z,\mathrm{NA}\right)=S_{\mathrm{amp}}\left(z,\mathrm{NA}\right)\cos\left(2\pi f_{B}\frac{z}{\nu }\right)e^{-z\alpha_{0}}$$

as shown in Fig. 7, here the simulated TRBS signal agree well with the experimental (||ΔR/R||) results presented in figure 5.

In all experimental and simulated traces, there is a clear attenuation in intensity of the TRBS signal as a function of depth (*z*). Aside from the effects of material attenuation (*α*_0_), it is reasonable to assume that a heavily diffracting sound field would have a detectable effect on the signal amplitude. However, it is clear from simulation (Fig. 3) and experiment (Fig. 5), that apparent attenuation appears to be dominated by an alternative effect: opto-acoustic defocus. The lack of contribution by acoustic diffraction can be understood by considering the case of optical plane wave detection (low probe NA) and a heavily diffracting sound field (high pump NA):

*Insidethe acoustic focal volume:* there are no evanescent components and therefore no near field diffraction, hence sound amplitude and optical intensity remain constant.

*Outsidetheacoustic focal volume:* acoustic diffraction starts to occur, hence sound amplitude decreases proportional to z. However, since the illumination is approximately collimated and constant, the TRBS signal remains constant. This is because with a low NA optical system, only the light that interacts with the area of the acoustic wavefront that is flat (area of the first Fresnel zone, *F*_0_) reaches the detector and contributes to the signal. As the area of this zone increases proportional to z, the signal loss due to acoustic diffraction is compensated. A figure of merit for this effect on the TRBS signal can be described with *TRBS* ∝ *F*_0_ * *U*_*sound*_ ∝*z* * *z*^−1^. Furthermore, this condition is only valid while *F*_0_ is smaller than the area of illumination; however, in experimental practice, material attenuation dominates.

In practice, it is difficult to observe the effects of acoustic diffraction using TRBS, as it would require a small yet collimated probe spot, and a larger diffracting acoustic spot; circumstances which are contradictory within the Bragg condition.

For the case of opto-acoustic defocus, the problem can be described within the context of geometrical optics: if the acoustic wavefront is considered an object in an infinity corrected microscope, as that object moves its image will no longer converge at the detector plane. As higher NA lenses are used, this deviation of the image plane will magnify. Within a TRBS trace, the effect of opto-acoustic defocus can be evaluated by comparing the Rayleigh range of the probe objective with the propagation distance of the signal (as seen in Fig. 3a).

In an imaging context it is often desirable to maximise resolution, however this requires increasing the NA of the objective lens thereby increasing apparent attenuation. Using the methodology presented in this report, the errors introduced by apparent attenuation can be removed by first modelling *α*_*def*_ and subtracting it from the experimental measurement ($\alpha_{\mathrm{def}}^{\prime }$) to recover *α*_0_ (see equation 5). Despite being able to compensate for the error on measuring *α*_0_, the ability to measure the Brillouin frequency in-depth will still be compromised by opto-acoustic defocus.

Alternatively, a better compromise between resolution an imaging depth can be achieved by a mismatched pump-probe NA configuration: maximise resolution (high NA_*pump*_) while minimising opto-acoustic defocus (low NA_*probe*_, see Fig. 6). However, this compromises signal-to-noise ratio (SNR) due to the following reasons: the effective cross section at which pump and probe spots interact is significantly reduced, the drop in power density due to enlarging spots and the difficulty of alignment of opposing objectives.

**Fig. 8:**
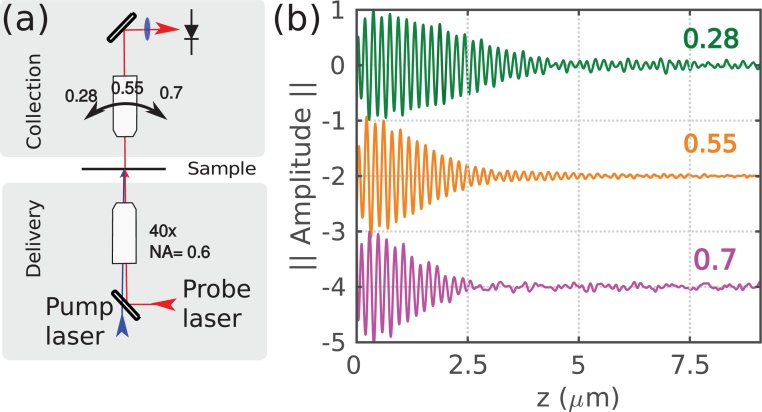
Alternative configuration of phonon microscopy. By reducing the NA of the collection lens it is possible to reduce the apparent attenuation by opto-acoustic defocus. (a) Shows the experimental setup: delivery NA remains fixed so that the resolution available for imaging remains constant as it is governed by the pump spot size. (b) Resulting TRBS signals obtained with different collection: the lowest NA shows longer lasting signals however resolution remains the same, leading to a better compromise between imaging depth and resolution compared to matched NA.

In previous works [9], [12], we have proposed an alternative optical arrangement (presented in Fig. 8) where one objective (high NA) is used to deliver pump and probe light and a second objective (low NA) is used to collect probe light in transmission. Preliminary experiments show this minimises the effects of opto-acoustic defocus (Fig. 8b), while also simplifying alignment and optimising the power density of the probe for a constant average input power. In this configuration, probe light which originates from a high NA objective, and is collected with a low NA objective, will suffer a reduction in SNR in the following way: the highest probe spatial frequencies will have angles that are beyond the light-cone of the low NA objective and will miss the detector. Overall the trade-offs offered by this configuration enhances the applicability of phonon microscopy, however, further modelling and optimisation are required.

## 5 Summary

We have isolated, in simulation and experiment, the effects of acoustic diffraction and opto-acoustic defocus in TRBS signals as NA increases. By doing so, we have identified *opto-acoustic defocus* as a strong cause of premature decay of TRBS signals which we call *apparent attenuation*. By understanding the mechanisms of signal decay, we have removed ambiguities and errors related with the NA of the system allowing us to accurately measure the sound attenuation coefficient. Additionally we proposed an alternative experimental configuration for phonon microscopy which offers a better compromise between resolution and imaging depth. This opens the possibility to use the sound attenuation coefficient as an additional contrast mechanism which could aid in identifying new biomarkers in cell biology.

## Funding

This work was supported by the Engineering and Physical Sciences Research Council [grant number EP / K021877/ 1, EP / G061661/1]; the Royal Academy of Engineering under the Research Fellowships scheme [RF_201718_17144].
