# Supplementary material for: Apparent attenuation by opto-acoustic defocus in phonon microscopy
Source: Photoacoustics. 2020 May 17;19:100180. doi: 10.1016/j.pacs.2020.100180 (PMC7262445; doi:10.1016/j.pacs.2020.100180)
Supplement: Supplementary file 1 [file mmc1.pdf]

Supplementary Material for “Apparent attenuation by optical defocus in phonon microscopy”

Fernando Pérez-Cota, Salvatore La Cavera III, Shakila Naznin, Rafael Fuentes-Dominguez, Richard J. Smith and Matt Clark

April 30, 2019

This document presents additional information regarding “Apparent attenuation by optical defocus in phonon microscopy”. Here some alternative simulation results are presented along with some examples of data processing.

Gaussian and Airy disks

Figure 1 shows an example of the output modulation depths S_{amp} from the simulation, using Gaussian optical fields. Compared to the simulations obtained using the Airy disk representation of the optical fields (used in the main manuscript), these do not show high frequency modulation at high NA. This modulation arises from the changes in phase at the Airy disk rings, whereas the phase in the Gaussian case are flat. The low frequency roll-offs remain similar. In the experimental results shown in figure 5a of the manuscript, the high frequency modulation can also be seen for the 0.7 NA (at approximately $z_r = 6\mu$m). In order to incorporate these high frequency components in the model, the Airy disk representation of the optical field was chosen.

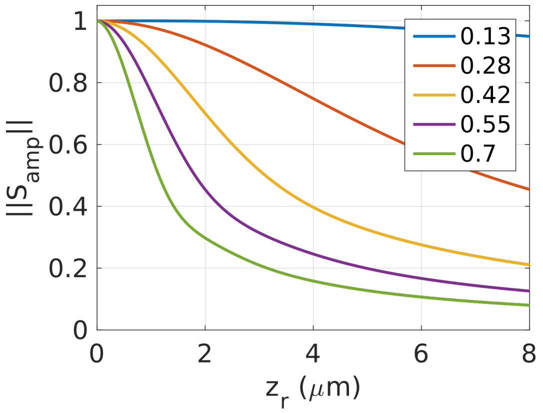

Figure 1: Simulated results of modulation depth S_{amp}, attained using Gaussian optical fields. These results are analogues to those shown in figure 3a in the main manuscript. Compared to the use of Airy disks, there are no high frequency modulations yet the roll-offs are similar. NA is specified in legend box.

Water and Glass

In order to validate the applicability of the findings of the main manuscript to water or aqueous solutions (such as cell media), we present simulated results (S_{amp}) using water as the external medium as opposed to glass (see Figure 2). The results are very similar to those of glass, shown in the manuscript figure 3a, since the acoustic wavelengths between water and glass only differ by the change in refractive index (1.45,1.33 for glass and water respectively):

$$\Delta\lambda_{sound} = \lambda_{probe}/2\Delta n \tag{1}$$

where Δn is the difference between the refractive index of the materials and λ_{probe} the optical probing wavelength. Even though the acoustic frequency differs by a factor of four, due to the difference in sound velocity from the two materials, the probed acoustic wavelengths remain similar ($\sim$10% variation). In this scenario, the material attenuation will be different however, apparent attenuation is similar.

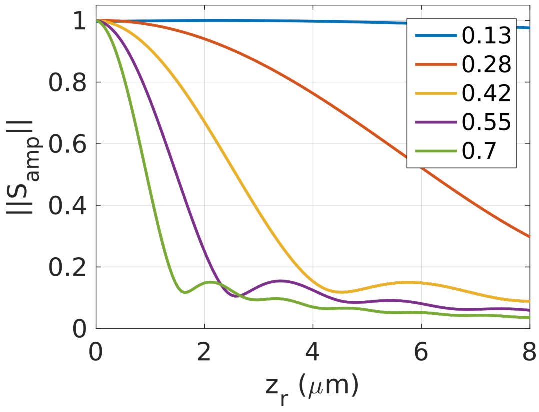

Figure 2: Simulated results of modulation depth S_{amp}, attained using water n=1.33 instead of glass. This result is similar to that of figure 3a from the main manuscript because the difference between refractive indexes is small.

Fitting

Exponentials were fitted to the simulation outputs, i.e. the decaying modulation depths (S_{amp}). The fitting is an exponential approximation which is sufficient for comparing the relative decay rates within the context of the experimental findings: the signal decay is a combination of apparent (defocus) and material attenuations. Figure 3 shows examples of these fittings where the match is good.

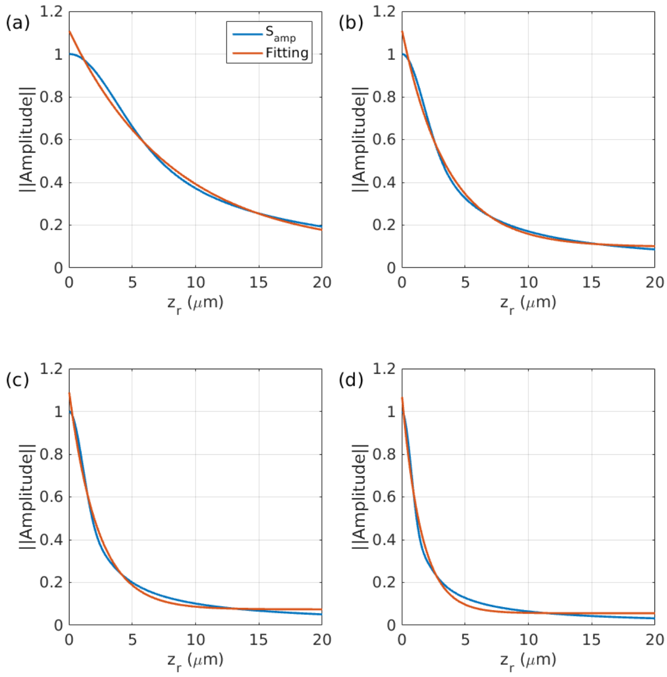

Figure 3: Examples of fitting process used to produce figure 6 from the main manuscript. NA= 0.28(a), 0.42(b), 0.55(c) and 0.7(d).
